# bia-binder: a web-native cloud compute service for the bioimage analysis community

**DOI:** 10.1093/bioinformatics/btaf412

**Published:** 2025-07-22

**Authors:** Craig T Russell, Jean-Marie Burel, Awais Athar, Simon Li, Ugis Sarkans, Jason Swedlow, Alvis Brazma, Matthew Hartley, Virginie Uhlmann

**Affiliations:** European Molecular Biology Laboratory, European Bioinformatics Institute (EMBL-EBI), Cambridge CB10 1SD, United Kingdom; School of Life Sciences, University of Dundee, Dundee DD1 4HN, United Kingdom; European Molecular Biology Laboratory, European Bioinformatics Institute (EMBL-EBI), Cambridge CB10 1SD, United Kingdom; School of Life Sciences, University of Dundee, Dundee DD1 4HN, United Kingdom; European Molecular Biology Laboratory, European Bioinformatics Institute (EMBL-EBI), Cambridge CB10 1SD, United Kingdom; School of Life Sciences, University of Dundee, Dundee DD1 4HN, United Kingdom; European Molecular Biology Laboratory, European Bioinformatics Institute (EMBL-EBI), Cambridge CB10 1SD, United Kingdom; One Health Informatics, Biomedical Research and Study Centre, Riga LV-1067, Latvia; European Molecular Biology Laboratory, European Bioinformatics Institute (EMBL-EBI), Cambridge CB10 1SD, United Kingdom; European Molecular Biology Laboratory, European Bioinformatics Institute (EMBL-EBI), Cambridge CB10 1SD, United Kingdom; Department of Molecular Life Sciences, University of Zurich, 8057 Zurich, Switzerland

## Abstract

**Summary:**

We introduce BioImage Archive Binder (bia-binder), an open-source, cloud-architectured, and web-based coding environment tailored to bioimage analysis that is freely accessible to all researchers. The service generates easy-to-use Jupyter Notebook coding environments hosted on EMBL-EBI’s Embassy Cloud, an academically hosted compute service which provides significant computational resources. The bia-binder architecture is free, open-source and publicly available for deployment. It features fast and direct access to images in the BioImage Archive, the Image Data Resource, and the BioStudies databases. We believe that this service can play a role in mitigating the current inequalities in access to scientific resources across academia. As bia-binder produces permanent links to compiled coding environments, we foresee the service to become widely used within the community and enable exploratory research.

**Availability and implementation:**

bia-binder is built and deployed using helmsman and helm and released under the MIT licence. It can be accessed at binder.bioimagearchive.org and runs on any standard web browser.

## 1 Introduction

In recent years, the variety, complexity and volume of biological images has increased exponentially, translating into an ever-growing need for sophisticated image analysis (Bagheri *et al.* 2022). Deep learning (DL), a subset of artificial intelligence, has been instrumental in addressing the “big data” challenge of bioimaging and has become the *de facto* standard for most of the classical bioimage analysis tasks (Meijering 2020). In particular, the ability of DL-based approaches to automatically recognize objects and extract relevant features from images is well underway to revolutionize how bioimages are analyzed, from fundamental biology research (Hallou *et al.* 2021) to biomedicine (Wainberg *et al.* 2018). The potentially game-changing capabilities of modern bioimage analysis methods however still remain out of reach for most researchers without a strong background in computer science. Due to the size of modern microscopy data, handling and processing them indeed often requires advanced scientific computing expertise—even in situations where the computing power needed by the analysis algorithms *per se* is reasonable. This accentuates the already-existing problem of unequal access to compute resources, data, and know-how. As researchers wanting to adopt DL in their analysis require extensive computational memory as well as fast network access to image data stores, an accessibility barrier stands between the computational resources needed to address contemporary medium-to-large-scale image analysis and the now widely available methods for that purpose. A growing divide separates those who are fortunate enough to have access to computational resources and those who do not, raising concerns around fairness in access to DL-methods for bioimaging.

A number of tools have been developed toward the goal of providing easy-to-use and accessible state-of-the-art bioimage analysis methods. In addition to well-established desktop applications such as Fiji (Schindelin *et al.* 2012), napari (Chiu *et al.* 2022), and QuPath (Bankhead *et al.* 2017), new web-based platforms have recently been developed. Among them, ImJoy (Ouyang *et al.* 2019) offers a browser-accessible computational platform that provides a user-friendly interface for developing and sharing image analysis workflows. ZeroCostDL4Mic (Chamier *et al.* 2020) and its evolution DL4MicEverywhere (Hidalgo-Cenalmor *et al.* 2024) adopt the different strategy of democratizing advanced bioimage analysis by providing a carefully curated no-code, self-explanatory interface to interact with deep learning-based tools for various image analysis tasks, including popular methods such as multi deep-CARE (Weigert *et al.* 2018) for image restoration and StarDist (Schmidt *et al.* 2018) for image segmentation. These various platforms are highly valuable to address the method accessibility challenge of bioimage analysis, but are limited in their ability to scale to large datasets or to provide access to high-performance computing resources.

To address these limitations, we have implemented a cloud computing service that specifically aims to alleviate the resource availability gap in the research community. The BioImage Archive Binder, bia-binder for short, is an open-source, cloud-based, and web-native coding environment that is freely accessible to any researcher. The bia-binder service is built around the Binder and JupyterHub ecosystems, which provide extensive documentation for beginners (see https://the-turing-way.netlify.app/communication/binder/zero-to-binder.html and https://jupyter-notebook.readthedocs.io/en/latest/examples/Notebook/examples_index.html, among many others). The service generates Jupyter notebook coding environments (Kluyver *et al.* 2016) that are ubiquitous in both the data science and teaching worlds, and that facilitate the quick prototyping of data analysis workflows. Jupyter notebooks are in particular the tool of choice for Python-based bioimage analysis at any proficiency level, and their uptake by non-computational researchers is supported by many freely available training resources (see https://haesleinhuepf.github.io/BioImageAnalysisNotebooks/, https://github.com/guiwitz/PyImageCourse_beginner, and https://github.com/uhlmanngroup/imagequantification101/ among many others). In the spirit of open science, environments within the service are shareable, deterministic, and compliant with modern FAIR standards for data analysis (Wilkinson *et al.* 2016).

## 2 bia-binder

The bia-binder (binder.bioimagearchive.org) is a cloud-hosted web-service that allows users to directly convert public code-repositories from public hosting services such as GitHub (https://github.com/) and Zenodo (https://zenodo.org/) into interactive Jupyter notebooks through BinderHub (https://binderhub.readthedocs.io/). BinderHub builds deterministic environments using repo2docker (https://repo2docker.readthedocs.io/) and deploys them on Kubernetes clusters (https://kubernetes.io/) as containers running Jupyter. The repo2docker library provides a flexible set of buildpacks that can work with several environment management tools such as conda, pip, apt, and Docker files.

In addition to the base bia-binder that can be used by anyone without login, we also provide a login portal for institutional users through Elixir-AAI (https://elixir-europe.org/services/aai), where additional resources including more RAM and CPU cores are provided (login.binder.bioimagearchive.org). The login portal links to JupyterHub (https://jupyter.org/hub), a gateway provider for generating online Jupyter notebooks which give access to common programming environments in notebook form, such as Python, Julia, R, along with Octave and Java-like languages through BeakerX (http://beakerx.com/). JupyterHub also includes Dask (Gueroudji *et al.* 2021) access, which is especially convenient for tasks involving larger-than-memory image data (a notebook demonstrating the use of BioImage Archive data stored in the OME-Zarr format and analyzed using Dask can be found at https://github.com/ome/EMBL-EBI-imaging-course-04-2024/blob/main/Day_5/Conversion.ipynb). While bia-binder can automatically and conveniently convert data and code repositories into Jupyter notebook environments, the JupyterHub gives authenticated users the option to access additional resources as well as a small permanent storage area (limited to 10GB for one year) for installing custom programming environments and packages alongside the Python, R, Fiji, OMERO (Allan *et al.* 2012) and Julia (Bezanson *et al.* 2017) environments already provided. We anticipate that the CPU and memory resources provided by bia-binder, even to unauthenticated users, will be above those available on a basic laptop and will as a consequence allow running DL-based approaches in inference mode at enhanced speed. The bia-binder service however does not provide dedicated access to GPU resources and is therefore not meant to be used for DL model training.

**Figure 1.**
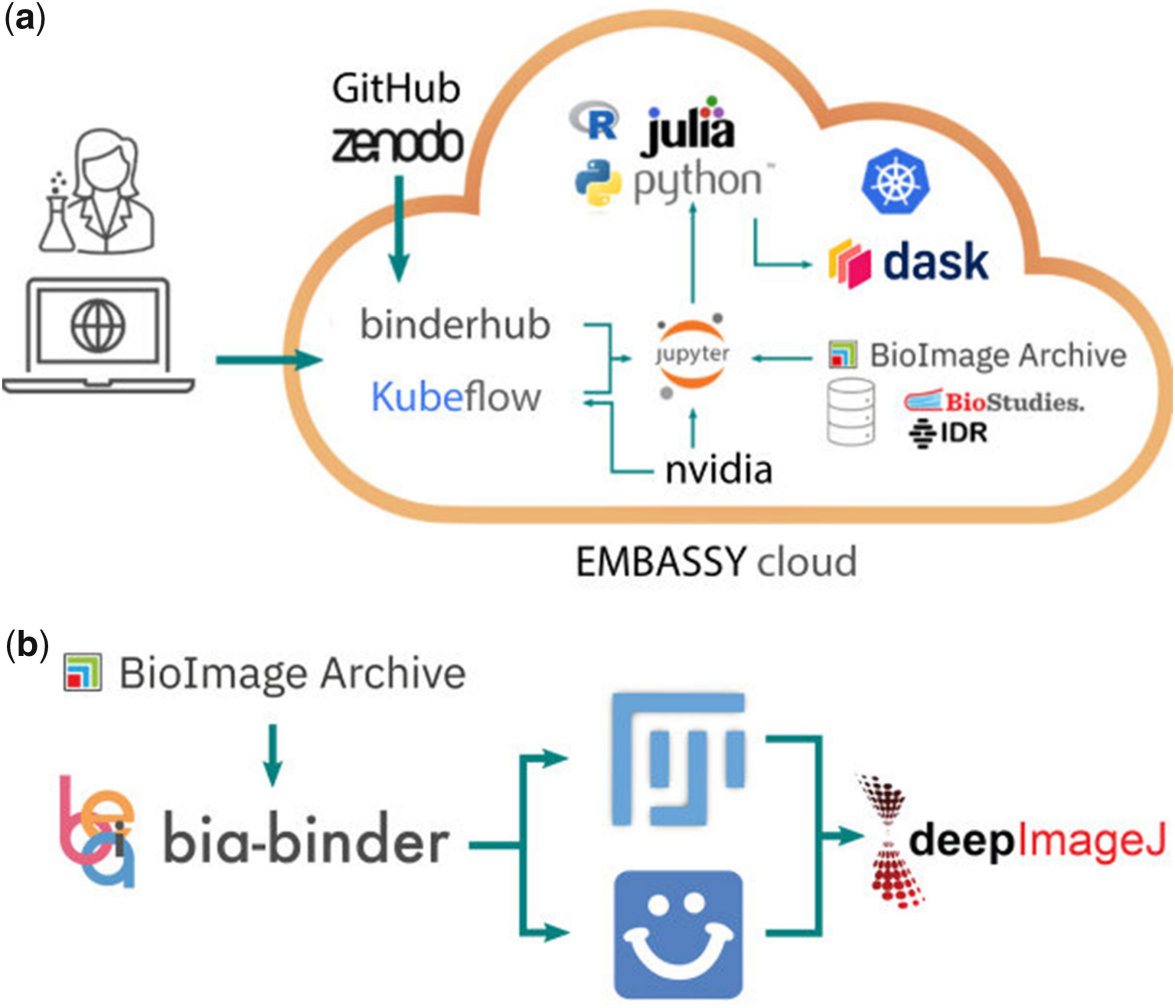
The bia-binder is deployed on Embassy Cloud hosted at EMBL-EBI. (a) It connects web-based Jupyter environments to the BioImage Archive, giving access to any Jupyter-compatible coding environment, to computational resources, and to analysis scaling tools such as Dask. (b) The bia-binder also readily integrates other available tooling ecosystems such as ImJoy and Fiji.

In Fig. 1, we illustrate how BinderHub and JupyterHub interact within the bia-binder environment and how bia-binder integrates with other tools in the bioimage analysis ecosystem. As Imjoy is a web-based platform, it can be directly interacted with through Jupyter (the examples provided and managed by the ImJoy team at https://github.com/imjoy-team/imjoy-jupyter-extension and https://github.com/imjoy-team/imjoy-hybrid-desktop can readily be used in bia-binder) and is also installed as a *de facto* plugin for authenticated users. Users can also interact with Fiji and the extended ImageJ ecosystem, including deepImageJ, either programmatically in Python through the pyImageJ library (Schindelin *et al.* 2012), or using the classical GUI through an ImJoy hybrid desktop environment.

We are hosting a free, public instance of bia-binder on the EMBL-EBI Embassy Cloud (https://www.embassycloud.org/), a service that provides significant and scalable computational resources for data analysis activities alongside EMBL-EBI’s public datasets. As such, the Embassy Cloud is collocated with, and directly linked to, the BioImage Archive (Hartley *et al.* 2022), the Image Data Resource (Williams *et al.* 2017), and BioStudies (Sarkans *et al.* 2018). The bia-binder thus has fast and direct access to several terabytes of publicly available reference image datasets. Importantly, user authentication only affects the computational resources available: all users, regardless of whether they are logged in or not, have access to the same bioimage data from these public repositories and to the same programming environments. Authenticated users, however, are granted storage space when using the service and therefore have the additional privilege of persistent configurations, which is closer to what they would experience if they were running Jupyter notebooks locally on their personal computer. This means in particular that unauthenticated users can only run public notebooks, and that notebooks stored on authenticated user accounts can only be viewed and edited by that authenticated user. While we chose to keep the starting environment as minimalistic as possible for the sake of flexibility, additional libraries such as ome_zarr or dask can be installed in different ways. Most straightforwardly, users can install them directly in a live session after launching an example environment (for instance from one of our example notebooks) using pip install. While it is generally advised against leaving pip install cells in notebooks, they can be very useful for debugging. More advanced users are encouraged to store their notebooks publicly relying on services such as GitHub or Zenodo and include a requirements.txt or an environment.yaml file, which will be automatically detected by the build system and lead to the installation of any additional dependencies when loaded in bia-binder. Finally, authenticated users can also create persistent environments to permanently store their dependencies.

Additional user-specific image data may be ingressed through standard file transfer protocols enabled here with RClone (https://rclone.org/), installed natively on bia-binder’s login-only JupyterHub services. Private storage areas are encrypted, no user authentication passwords are stored on the cluster, and user activities and data are neither tracked nor stored. The bia-binder backend Kubernetes deployment is managed by Embassy Cloud and regularly maintained and updated for vulnerabilities. For both anonymous and logged in users, active sessions are respected for up to a week before being culled and inactive sessions are given a 12 h lifetime to ensure that resources are freed up for other users. BinderHub allows users to build notebooks from tags and branches on GitHub and therefore provides version control. The environments built by Binder are also highly consistent due to hard requirements on dependency versions and to the underlying use of Docker through repo2docker. As a result, the bia-binder environments are extremely reproducible.

The bia-binder deployment codebase is open-source and publicly available at https://github.com/BioImage-Archive/bia-binder under the MIT license. The bia-binder architecture is implemented to be cloud-provider agnostic and is therefore amenable to further deployment on other computational infrastructures that support Kubernetes.

The instance of bia-binder hosted at EMBL-EBI is solely reliant on Embassy Cloud being a persistent service. Further deployments are however facilitated by the fact that bia-binder does not depend on any private resources as Kubernetes, Jupyter, and JupyterHub are fully open-source. Furthermore, we designed bia-binder to comply with FAIR data principles in that analysis pipelines are

- *Findable*, as BinderHub creates permanent links to data-pipelines that are stored as notebooks (.ipynb) on public repositories (access to these BinderHub images can then, in-turn, be assigned a DOI using public archiving services such as Zenodo);
- *Accessible*, as the bia-binder service is free and publicly available;
- *Interoperable*, as Jupyter provides native interoperability through its web-based nature and uses standard API calls and HTML, and as analysis environments are built on Docker containers, making them natively interoperable through Open Container compliance (https://opencontainers.org/);
- *Reusable*, as documentation, example scripts and environments for interfacing with public bioimage databases such as the BioImage Archive are provided to support users in applying gold-standard analysis methods to new and archived data alike.

## 3 Use-cases

We primarily expect bia-binder to be interacted with as a teaching, training, and data exploration tool to carry out bioimage analysis in a convenient programming environment with attached resources. One concrete use-case where bia-binder contributes to enhancing accessibility is by allowing researchers to interact through Jupyter notebooks with image datasets that would otherwise be hard to handle due to their size or scale. This is typically the case for drug screens, as found in the IDR (see Table 1 for specific examples): although individual images in these datasets may not be large on their own, an entire dataset can easily reach the terabyte scale. Interacting with these outside of bia-binder would therefore necessitate downloading terabytes of images and processing them locally, which requires a network bandwidth and stability as well as memory resources that are far from granted for many researchers. With the help of bia-binder, everyone is empowered to computationally browse such datasets to spot check which images or features they may be interested in analyzing further. From there, it then becomes possible to identify a subset of files to download, and to carry out a focused analysis locally. To illustrate this, we provide several example notebooks that demonstrate how data from the IDR, the BioImage Archive, and BioStudies can be accessed through bia-binder in our documentation at https://bia-binder.readthedocs.io/en/latest/, made easily accessible via the *readme* button on the bia-binder website. The stable examples provided under “quick start” demonstrate specific functionalities such as accessing images from Biostudies or creating an ImJoy Hybrid Desktop and a Visual Studio Code environment. We also point to contributed third-party notebooks, marked as “experimental features,” that illustrate further use-cases. These experimental notebooks are however not directly maintained by us and may as a result require manual adjustments.

**Table 1.** Examples of large IDR screens accessible through bia-binder.

Experiments	Image dataset size	Associated metadata
IDR0006	16.6 TB	Genes (localisation)
IDR0036	1.20 TB	Cell states
IDR0093	1.62 TB	Genes (morphometric readouts)
IDR0094	1.41 TB	COVID Drug response

As it demonstrates the benefit of having fast access to substantial amounts of publicly available data, bia-binder also showcases the value of bioimage data sharing and has thus the potential to contribute to a broader shift toward a more open research culture in bioimaging.

## 4 Conclusions

We have developed bia-binder to provide a convenient and capable platform for performing bioimage analysis with the wide range of cutting-edge open-source algorithms on the large corpus of publicly available microscopy image datasets, which is growing exponentially. A promising future direction of work is the inclusion of tools for online, collaborative, and versioned image annotation in services such as bia-binder, toward which the recently launched Kaibu (https://kaibu.org/) provides a first attempt. Although we do not envision bia-binder to replace commercial cloud providers, it offers a rare academically hosted compute resource alternative. We believe that bia-binder will contribute to mitigating the current inequality in access to advanced bioimage analysis across the globe, which is chiefly driven by lack of access to computational resources. Furthermore, as bia-binder provides permanent links to compiled coding environments through BinderHub, we foresee the service to be widely adopted by the community as a way to share interactive examples of algorithms and analyses. Given that provision for Kubernetes cloud environments is becoming more widely available, it is possible for this service to be mirrored across multiple institutes with a single gateway federating access to the partners’ deployments for analysis tasks that do not need direct access to EMBL-EBI-hosted image databases.

## Data Availability

No new data were generated or analysed in support of this research.
